# Review of the continental Oriental species of *Lilioceris* Reitter (Coleoptera, Chrysomelidae, Criocerinae) closely related to *Lilioceris impressa* (F.)

**DOI:** 10.3897/zookeys.103.983

**Published:** 2011-06-10

**Authors:** Alexey K. Tishechkin, Alexander S. Konstantinov, Sanjay Bista, Robert W. Pemberton, Ted D. Center

**Affiliations:** 1 Department of Invertebrate Zoology, Santa Barbara Museum of Natural History, 2559 Puesta del Sol Rd., Santa Barbara, CA 93105 USA; 2 Systematic Entomology Laboratory, USDA, c/o Smithsonian Institution P.O. Box 37012 National Museum of Natural History, MRC-168 Washington DC 20013-7012 USA; 3 Division of Entomology, Nepalese Agricultural Research Council, Khumaltar Katmandu, Nepal; 4 USDA-ARS, Invasive Plant Research Laboratory, 3225 College Ave., Fort Lauderdale, FL 33312

**Keywords:** Coleoptera, Chrysomelidae, Criocerinae, *Lilioceris*, key, Oriental Region, new synonyms, *Dioscorea bulbifera*, air potato, biology, *Lilioceris cheni*

## Abstract

Criocerine leaf beetles found in Nepal feeding on *Dioscorea bulbifera* (L.), an invasive weed of Asian origin, were identified as *Lilioceris cheni* Gressitt and Kimoto based on a synopsis of the Oriental *Lilioceris* species and review of the *Lilioceris impressa* species group. All the continental, Oriental species included in the group are diagnosed and illustrated, and a key for their identification is provided. Species status of *Lilioceris thibetana* Pic, 1916 is resurrected. The following new synonyms are proposed: *Lilioceris coomani* (Pic, 1928) = *Lilioceris egena* (Weise, 1922), and *Lilioceris subcostata* (Pic, 1921a), *Lilioceris laticornis* (Gressit, 1942), *Lilioceris inflaticornis* Gressit & Kimoto, 1961, and *Lilioceris maai* Gressit & Kimoto, 1961 = *Lilioceris impressa* (Fabricius, 1787). Lectotypes of the following species are designated: *Lilioceris coomani* Pic, 1928; *Lilioceris impressa* (Fabricius, 1787); *Lilioceris laosensis* (Pic, 1916); *Lilioceris malabarica* (Jacoby, 1904); *Lilioceris ruficornis* (Pic, 1921b); *Lilioceris subcostata* (Pic, 1921a); *Lilioceris thibetana* (Pic, 1916); and *Lilioceris unicolor* (Hope, 1831).

## Introduction

This study was initiated by the need to identify leaf beetles that were feeding on *Dioscorea bulbifera* L.(Dioscoreaceae) in Nepal. *Dioscorea bulbifera* (air potato) is a herbaceous, perennial twining vine that attains lengths of 20 m or more, rendering it capable of climbing over and smothering native vegetation (e.g., Schmitz et al. 1997; Langeland and Craddock Burks 1998; Gordon et al. 1999). The species was introduced to Florida from tropical Asia or Africa in 1905 (Morton 1976). By the 1980s, air potato vines were growing in thickets, waste areas, and hedges or fencerows in many parts of south and central Florida (Bell and Taylor 1982). By 1999, *Dioscorea bulbifera* was recognized as an invasive exotic capable of altering plant communities by displacing native species and altering community structure and ecological functions (FLEPPC 2003). Vegetative propagation occurs primarily through aerial bulbils (hence the name “air potato”) that form in leaf axils during late summer, and may weigh up to 1 kg. These bulbils drop to the ground during the cooler months when the vines die back. Vines resprout during spring from subterranean tubers or from bulbils. Seed production is unknown in Florida and spread occurs mainly through anthropogenic dispersal of the bulbils (Schultz 1993). In order to find natural enemies of air potato, field explorations were conducted in Nepal and other Asian countries (Wheeler et al. 2007).

Leaf beetles that were found feeding on air potato in Nepal were not identifiable to species using existing keys (Gressit and Kimoto 1961, Kimoto and Gressit 1979), which are generally incomplete and often use unreliable characters. There are about 110 species of *Lilioceris* in the Oriental Region, about 80 of which occur on the continent. Synoptic study involving examination of the original descriptions and major leaf beetle collections (see list below) allowed us to identify a set of features that separated a relatively small group of species, including a species in question that is a potential air potato biocontrol agent, from the rest of the continental, Oriental *Lilioceris*.We refer to this group as the *Lilioceris impressa* species group and treat it in this paper, providing distinguishing characters of the group, a key to its species, and illustrations of characters, including the internal sac of the aedeagus.

## Material and methods

Specimen observation and preparation follow the methods of Konstantinov (1998). Specimens were examined from the following collections:

BBMBernice Bishop Museum, Honolulu, HI

BMNHThe Natural History Museum London, United Kingdom

CASCalifornia Academy of Sciences, San Francisco, CA

HUBMuseum für Naturkunde, Berlin, Germany

MNHNMuséum National d’Histoire Natural, Paris, France

UCDZoological Museum, University of Copenhagen, Copenhagen, Denmark

USNMNational Museum of Natural History, Smithsonian Institution, Washington, DC

ZMUHZoologisches Intsitute und Zoologisches Museum, Universität Hamburg, Hamburg, Germany

The most reliable characters for discrimination of *Lilioceris* species are those of the internal sac of the aedeagus. In Criocerinae, these characters were used previously for separation of *Lilioceris* species from Iran (Berti and Rapilly 1976) and *Oulema* species related to *Oulema melanopus* (L.) (Berti 1989). Terminology for the sclerites of the internal sac is not overly complicated. Berti and Rapilly (1976) called the longer, dorsally situated structure in the everted internal sac, the “pièce terminale”. We recognize three major sclerites in the internal sac of the aedeagus: dorsal [=“pièce terminale” of Berti and Rapilly (1976)], median, and ventral (Fig. 23). All these three parts differ from species to species within the *Lilioceris impressa* group, but the most reliable and easily observed characters are those of the dorsal sclerite.

Dissection and preparation of the sclerites of the internal sac of the aedeagus are relatively simple. We used slightly bent #1 entomological pins to extract the internal sac from the dorsal opening of the aedeagus that was soaked in hot 10% KOH solution for 15–20 minutes and washed in excess of water. Figures were generated using a Camera Lucida attached to a Zeiss Stemi SV 11 dissecting microscope. They were scanned and edited with Adobe Photoshop.

## Systematics of *Lilioceris impressa* species group

Species of the *Lilioceris impressa* group share the following characters: 1) glabrous scutellum almost completely lacking setae, with only a few setae occasionally present near the base (Fig. 7); 2) antennomeres 5–10 distinctly flattened, quadrate or even transverse (antennomere 5 often slightly elongate) and covered with dense, short, appressed setae (in clear contrast with more basal antennomeres bearing longer, more erect and much sparser setae, not obscuring the view of glabrous cuticle) (Fig. 6); and 3) structure of the sclerites of the internal sac of the aedeagus. The dorsal and ventral sclerites are roughly plank-like in shape, the dorsal one being longer and more complex shaped, especially anteriorly where it bears two relatively long lateral processes that vary in shape.

To a lesser extent color (traditionally often used in distinguishing and keying out *Lilioceris* species) is helpful to recognize representatives of the species group. Typically, they have a black head, thorax, abdomen and legs, and brownish-yellow to reddish elytra. Black legs and pale unspotted elytra are consistent throughout all species group members, while the head and thorax may be partially or entirely brown, reddish-brown or reddish; these paler colors are variably present around the abdominal apex.

The three characters mentioned above, in combination, define the group. As to the similar species, there are, on one hand, several similar looking black and red (yellow) species with a glabrous scutellum and similar structure of male genitalia, which have distinctly, often substantially elongate (never close to quadrate or transverse) antennomeres 5–10. On the other hand, a group of mostly entirely reddish species with the antennal characters identical to those of the *impressa*-group possess consistently a completely setose scutellum and differently shaped sclerites of the aedeagal internal sac (in particular, the dorsal sclerite is narrow, elongate and thread-like, at least apically). A few species with consistently spotted elytra (e.g., *Lilioceris bakewelli* Baly, *Lilioceris ruficollis* Baly) possess all of the characters in agreement with the *impressa*-group as it is outlined above.

Below, we are introducing several new synonymies and rearranging several previously proposed synonyms. There are two primary reasons for these changes in taxonomy of the Oriental *Lilioceris*. First, in the course of this study we introduced for the first time the use of male genitalia characters into the diagnostics of the species, in particular focusing on the sclerites of the internal sac of the aedeagus. In general, male genitalia morphology in the Oriental species appeared to be quite conservative. However, minor differences in the shape of the aedeagus and characters of the aedeagal sclerites of the internal sac are stable and consistent across vast species ranges and among multiple individuals within populations. This consistent set of aedeagal characters provides reliable diagnostics among species that are otherwise monotonous with respect to color and punctation. As a result, some misidentifications were corrected and species identities clarified. Second, the accepted concept of *Lilioceris impressa* (Gressit and Kimoto 1961, Kimoto and Gressit 1979), considered to be the most widespread and variable species, was found to be erroneous after the study of Fabricius’ authentic material. Contrary to the opinion of Gressit and Kimoto (1961) and illustrations in their monographs, the true *Lilioceris impressa* has an isolated oblique setose band on the posterior part of the outer metasternal disc (see Fig. 13c and Fig. 14c in Gressit and Kimoto 1961 and Kimoto and Gressit 1979, respectively). Consequently, their concept of *Lilioceris impressa* corresponds primarily to *Lilioceris laosensis*, with some *Lilioceris egena* specimens identified as *Lilioceris impressa* occasionally.

### Key to the species of *Lilioceris impressa*-group

**Table:** 

1	Outer parts of metasternal disc mostly free of setae; isolated latero-posterior setose patches or lateral extensions of anterior setose margins present in some species and widely scattered single setae may present on unworn individuals	3
-	Outer parts of metasternal disc or its lateral side mostly covered with dense setae; other characters variable	2
2	Internal sac of aedeagus with posterior part of dorsal sclerite in lateral view directed ventrally	*Lilioceris cheni* Gressit & Kimoto
–	Internal sac of aedeagus with posterior part of dorsal sclerite in lateral view directed dorsally	*Lilioceris unicolor* Hope
3	Outer parts of metasternal disc without isolated posterior setose patches	4
–	Outer parts of metasternal disc with isolated posterior setose patches; pronotum with scattered large punctures, elytral punctures not weakened posteriorly, elytral interval in pre-apical area distinctly convex	*Lilioceris impressa* (Fabricius)
4	Pronotal disc completely covered with scattered large punctures	5
–	Large punctures on pronotal disc present only as a row along mid-line, at least on anterior half, the rest of the disc utmost with only some tiny punctures	6
5	Elytral punctures on posterior third not weakened, more or less the same size and depth as on anterior half; pronotum posteriorly with single weak, regularly transverse impression; posterior part of dorsal sclerite of aedeagus in dorsal view directed laterally (Fig. 29)	*Lilioceris thibetana* (Pic)
–	Elytral punctures on posterior third variably, but distinctly, weakened; pronotum posteriorly with two weak, but distinct transverse irregular impressions; posterior part of dorsal sclerite of aedeagus in dorsal view directed medially (Fig. 27)	*Lilioceris laosensis* (Pic)
6	Occipital area with shallow furrow sometimes reduced to deep small fovea at midpoint; elytral intervals in preapical area flat	7
–	Occipital area without longitudinal furrow, only with weak impression and thin, indistinct suture; elytral intervals in preapical area distinctly convex	*Lilioceris egena* (Weise)
7	Anterior setose fringe of metasternum expanded on antero-lateral corner; lateral side of pronotum around constriction impunctate; most of metepisternal disc densely covered with setae	*Lilioceris malabarica* (Jacoby)
–	Anterior setose fringe of metasternum narrow, not expanded; lateral side of pronotum around constriction with several large punctures; setae on metepisternal disc present only as narrow strip along inner margin	*Lilioceris yunnana* (Weise)

## Species accounts

### Lilioceris cheni

Gressit & Kimoto, 1961

http://species-id.net/wiki/Lilioceris_cheni

**Figures 1–4.**
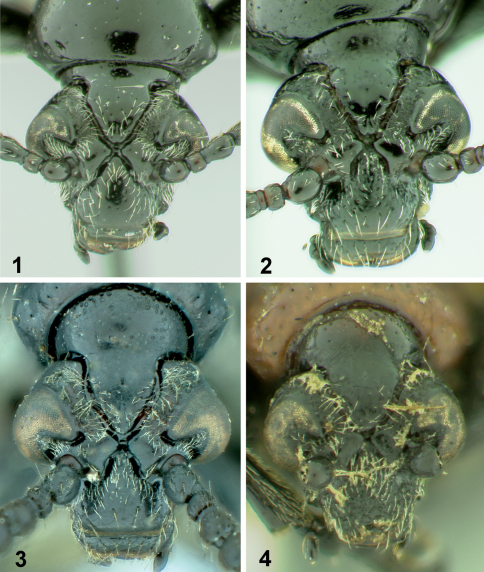
Heads of *Lilioceris* species, frontal view. **1** *Lilioceris cheni* **2** *Lilioceris egena* **3** *Lilioceris impressa* **4** *Lilioceris thibetana*.

Figs 1 6, 8 14, 21 23, 24

Lilioceris cheni Gressit & Kimoto, 1961: 46 (type locality: SE China Fukien, E. Kwangtung. Type depository: BBM).

#### Diagnosis.

 Occipital area with a shallow furrow, at least with a deep small fovea at mid-point. Apical elytral punctures strong. Pronotal disc with scattered larger punctures, mid-line alignment of punctures usually evident, at least in anterior half. Pronotum posteriorly with single weak, irregular, variable, transverse impression. Lateral sides of pronotum around constriction with large punctures. Outer metasternal disc almost completely covered with setae. At least half of metepisternal disc covered with setae. Apical elytral intervals distinctly raised. Internal sac of aedeagus with posterior part of dorsal sclerite in dorsal view more or less parallel-sided, directed medially. Posterior part of dorsal sclerite in lateral view more or less parallel-sided, bent ventrally, directed ventrally.

#### Comments.

 The holotype of *Lilioceris cheni* is housed in the Bishop museum collection. This collection contains 5 more specimens of this species all identified as *Lilioceris cheni* by Kimoto in 1967 and 1977, none of them are marked as paratype.

#### Biology.

 Pale white, oblong eggs of *Lilioceris cheni* are deposited in loosely aggregated clusters on leaves of its host plant, *Dioscorea bulbifera* (air potato: Dioscoreaceae). Females deposit, on average, more than 1200 eggs during their lifetime. The eggs become yellowish as the embryo develops and dark reddish eye spots appear mid-way through the incubation period. The entire incubation period requires about 4 days. The larvae are yellowish at first, becoming grayish in later instars, with black legs, head capsule, and prothoracic shield. They are often covered with a slimy substance to which fecal material adheres. Larvae feed gregariously and skeletonize the leaves from the underside. Young leaves are preferred but they also consume older, tougher leaves and are able to feed on the aerial bulbils. Complete development of the four larval instars requires about 8 days, with each instar lasting about 2 days each. When fully grown, larvae drop from the host plant to the soil which they quickly enter. They then produce a whitish oral exudate that hardens into a foam-like cocoon. Pupation often occurs gregariously, with several pupae clumped together within a matrix of this material. Adults emerge in about 16 days, begin mating in about 10 days following emergence, and initiate oviposition about 5 days later. The adults live 3 months or more and can survive a month without food.

In the Katmandu Valley of Nepal, the host plant drops its leaves during the cool, dry winter forcing the adult beetles to over-winter beneath debris on the ground. The adults emerge during mid-May to early June. Oviposition begins during late May and continues till mid-June. Females lay about 90 eggs/day during a 13-day period of ovipositional activity. Overwintered adult beetles live until mid-July, for about 76 days after emergence.

#### Type material examined.

 *Lilioceris cheni*: Holotype male: 1) Fukien, S. China, Shaowu city to Kaoyang, T. C. Maa Coll.; 2) July 30, 1945; 3) Holotype Lilioceris cheni J. L. Gressitt + K.; 4) L. cheni sp. n. Det. S. Kimoto (BBM).

#### Material examined.

 **CHINA.** **Yunnan:** Ma-Chang, 1000 m, 1 specimen (USNM); Tche-Ping-Tcheou, 1 specimen (USNM); 13–18.X.2010, RCVDIPRL 25. Oct. 10, M. Percell (USNM). **INDIA.** Indie orientales, 1 specimen (USNM). **Assam:** Chabua, 8.V.1944, G. Butler, 1 specimen (USNM). **LAOS.** **Sayaboury Prov.**, Sayabouri, 30.V.1965, native collector, 1 specimen (BBM); **Vientianne Prov.**, Vientianne, 21.IV.1965, J. A. Rondon, 1 specimen (BBM). **NEPAL.** On road to Dhunche, 5 km SW Ranipawa, 27°48.92'N, 85°13.41'E, 1700 m, 28.IV.2000, A. Konstantinov, S. Lingafelter & M. Volkovitch, 1 specimen (USNM); Gairigaonbelow Nagarkot,Kathmandu Valley, host *Dioscorea bulbifera*, 27.VIII.2002, Pemberton & Rayamhji, 1 specimen (USNM); Env.ofKathmandu, Palchoki Mount, 27°34.65'N, 85°24.04'E, 2300–2730 m, sweeping, 14.V.2000, A. Konstantinov, S. Lingafelter & M. Volkovitch, 1 specimen (USNM); Lalitpur, Kathmandu, 4.VIII.-15.X.2007, Yukawa & Junichi, 29 specimens (USNM); Seutikhola Dharan road, Sunsan, host *Dioscorea bulbifera*, 4.IX.2002, Pemberton & Rayamhji, 1 specimen (USNM); Tarai Reg., Narayangarh, along Rapti River, 27°42.31'N, 84°21.11'E, beating, 26.IV.2000, A. Konstantinov, S. Lingafelter & M. Volkovitch, 1 specimen (USNM).

### Lilioceris egena

(Weise, 1922)

http://species-id.net/wiki/Lilioceris_egena

Figs 2 9 15 25

Crioceris egena Weise, 1922:41 (Type locality: Fukien. Type depository, unknown).
Crioceris coomani Pic, 1928:88 (Type locality: Vietnam, Tonkin. Lectotype designated here, MNHN). New synonym

#### Diagnosis.

 Occipital area without longitudinal furrow. Pronotal disc without or with few small punctures, larger punctures present only along pronotal mid-line at least in anterior half. Lateral sides of pronotum around constriction with large punctures. At least half of metepisternal disc covered with setae. Outer metasternal disc mostly free of setae. Anterior setose fringes not expanded in antero-lateral corners.

Apical elytral punctures strong. Apical elytral intervals distinctly raised. Internal sac of aedeagus with posterior part of dorsal sclerite in dorsal view more or less triangular, directed forward. Posterior part of dorsal sclerite in lateral view more or less triangular, directed forward.

#### Comments.

 We were not able to find type material for this species. The type specimens were not found in Berlin (HUB) nor in Hamburg (ZMUH) (Peters, personal communication, August-September 2009). There is a strong possibility that the types were destroyed in Hamburg during World War II. However, two specimens identified as *Lilioceris egena* by J. Weise himself (including a male) were available for the study, and we based our concept of the species on these specimens. However, we are reluctant to designate the neotype at this moment, since an exhaustive search in other potential depositories has not been undertaken during the preparation of this manuscript. The male holotype of *Lilioceris coomani* perfectly corresponds to males of *Lilioceris egena* as we understand it now. This species is collected feeding on *Dioscorea subclava* Prain & Burkill in China.

#### Type material examined.

 *Lilioceris coomani*:Lectotype male [dissected on mounting cardboard] 1) [illegible] concave glabre; 2) 785 *Crioceris* sp.; 3) type; 4) TYPE; 5) coomani n sp; 6)Lectotype Lilioceris coomaniPic A. Tishechkin & A. Konstantinov des. 2010 (MNHN); paralectotype female on the same pin with “156” written on mounting cardboard; paralectotype designation label put on the same pin.

#### Material examined.

 **CHINA.** Kiang Li, Tengan, 2 specimens (USNM). **Anhui:** Taipingshien, X.1932, G. Liu, 1 specimen (USNM); **Yunnan**, Dali City,20.VIII.2010 (USNM). **Fujian:** Shaowu, Shui Pei Kai, T.C. Maa, 1 specimen (BBM); same locality and collector, but 25.III.1942, 1 specimen (USNM); without precise locality, G. Siemssen, 4.IX.1913, 2 specimens identified by J. Weise (USNM); with the same label, but 1.IV.1914, 1 specimen (USNM). **Hong Kong:** May 6 1940, P. K. To (1 BBM); Circa 1,600 ft, 24.IX.1937, Miss Harford. B.M. 1938-426 (1 BBM). Kieniyang Liutuan, T. C. Maa, 22.VIII.1942 (1 BBM). **Sichuan:** Shin Kai, 4400 feet, VIII.1922, D. C. Graham, 1 specimen (USNM); Wen-ch’uan, 4000–6000 feet, VII.1938, D. C. Graham, 1 specimen (USNM). **INDIA. Assam:** Darjeeling (Himalayas), 1 specimen (USNM). **Karnataka:** Mysore, 2 specimens (USNM). **Uttarhand:** Dehra Dun, 600 m, 18.VII.1973, G. Ekis, 2 specimens (USNM); 3 km NW Rishikesh, 10.V.1975, J. L. Petty, 1 specimen (USNM). **LAOS.** **Vientianne Prov.**, Ban Van Eue, 1–15.IX.1967, native collector, 1 specimen (BBM); Phou-kow-kuei N of Vientianne, 17.IV.1965, J. L. Gressit, 1 specimen (BBM). **NEPAL.** Env.of Kathmandu, Bonipa, 27º40’N, 85º25’E, 8.V.2000, A. Konstantinov, S. Lingafelter & M. Volkovitch, 1 specimen (USNM); Jiri**-**Kathmandu road, high pass between Sikri and Kabre (Mudee), 27º42’7”N, 85º56’24”E, 2632 m, V.2000, A. Konstantinov, S. Lingafelter & M. Volkovitch, 1 specimen (USNM); Tarai, env. Chitwan Park, 27º28.79’N, 84º52.54’E, 300 m, 25.IV.2000, beating, A. Konstantinov, S. Lingafelter & M. Volkovitch, 1 specimen (USNM); Tarai Reg., Narayangarh, along Rapti River, 27º42.31’N, 84º21.11’E, beating, 26.IV.2000, A. Konstantinov, S. Lingafelter & M. Volkovitch, 1 specimen (USNM). **SINGAPORE.** Without precise location, Coll. Baker, 19 specimens (USNM). **VIETNAM.** Saigon, 1 specimen (USNM); Trang Bom, 30 mi NW of Saigon, 18.VII.1932, M. Poilane, 1 specimen (USNM).

**Figures 5–9.**
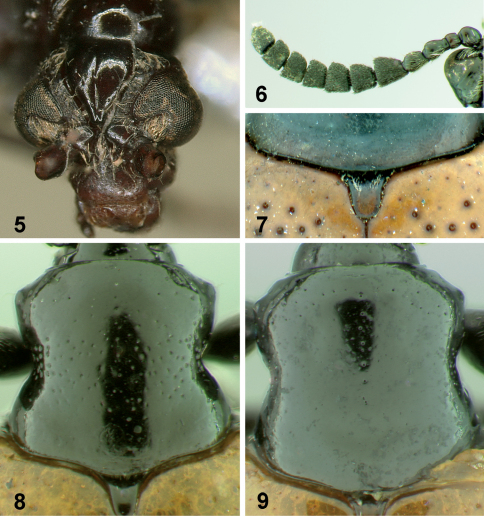
Body parts of *Lilioceris* species. **5** head of *Lilioceris unicolor*, frontal view **6** antenna of *Lilioceris cheni* **7** scutellum of *Lilioceris impressa;* **8–9** pronotum **8** *Lilioceris cheni* **9** *Lilioceris egena*.

### Lilioceris impressa

(Fabricius, 1787)

http://species-id.net/wiki/Lilioceris_impressa

Figs 3 7 10 16 26

Crioceris impressa Fabricius, 1787: 88 (Type locality: “Siam”. Lectotype designated here, BMNH).
Crioceris subcostata Pic, 1921a:2 (Type locality: China, “Shin-guey-fu”. Lectotype designated here, MNHN). New synonym
Crioceris ruficornis Pic, 1921b: 136 (Type locality: China, “Pe Yen Tsing (Yunnan)”. Lectotype designated here, MNHN). Gressitt and Kimoto 1961:59 (synonymy).
Crioceris laticornis Gressit 1942:300 (Type locality: Hainan, Kwantung. Type depository: unknown). New synonym
Lilioceris inflaticornis Gressit and Kimoto, 1961:50 (Type locality: “SE China [Kwantung, Fukien]. Type depository: CAS). New synonym
Lilioceris maai Gressit and Kimoto, 1961:53 (Type locality: “Ta-chu-lan, near Shaowu, NW Fukien”. Type depositary: BBM). New synonym

#### Diagnosis.

 Occipital area with shallow furrow, at least with deep small fovea at midpoint. Pronotum posteriorly with single weak, regular, transverse impression. Pronotal disc with scattered larger punctures, mid-line alignment of punctures usually still evident, at least on anterior half. Lateral side of pronotum around constriction with large punctures. Outer metasternal disc only with oblique setose patch. Anterior setose fringe not expanded in antero-lateral corner. At least half of metepisternal disc covered with setae. Apical elytral punctures strong. Apical elytral intervals distinctly raised. Internal sac of aedeagus with posterior part of dorsal sclerite in dorsal view elongate, narrowing posteriorly, directed slightly laterally. Posterior part of dorsal sclerite in lateral view widening and round at apex, directed forward.

#### Comments.

 All five species put here into synonymy with *Lilioceris impressa* correspond to it well. There is slight variability in the size of the outer metasternal setose patch, and color varies from almost completely reddish-brown to typical black and yellow, but pronotal and elytral punctuation and sculpture as well as aedeagus characters are consistent between specimens. We did not observe any meaningful variability in the shape of the expanded distal antennomeres, which was used by Gressit and Kimoto in describing *Lilioceris inflaticornis* and *Lilioceris laticornis*. However, even these authors had some doubts about the use of these characters, even in the reference to the type series (Gressit and Kimoto 1961).

We did not thoroughly search for *Lilioceris impressa*-group specimens originating from outside of the area of interest, in particular from the islands and archipelagoes of the Oriental Region. However, all the island specimens in the USNM identified as *Lilioceris impressa* do not belong to this species and all lack the outer metasternal setose patch. So, the prevailing concept of *Lilioceris impressa*, being widely distributed across almost the entire Oriental Region, seems to be at least questionable. All the USNM *Lilioceris impressa*-group specimens originating from Sri Lanka, Andaman Islands, Greater and Lesser Sunda Islands, and Philippines were not identified as any of the species dealt with in this study and might indeed represent undescribed species closely related to other *Lilioceris impressa*-group species.

#### Type material examined.

 *Lilioceris impressa:* Lectotype, male. Labels. 1) Cr. impressa Fabr. Mant. Ind. n. 24; 2) Sir Joseph Banks Collection 1743–1820 ex. Linn. Soc. 1863 BM(NH) 1863-46; 3) ‘Type’ Crioceris impressa Fab. 1787; 4) Lectotype Crioceris impressa Fabricius des Konstantinov and Tishechkin 2010 (BMNH).

*Lilioceris inflaticornis*: Paratype, male, Fujian: Gang-keu, 26.VII.1936, J. L. Gressitt, 1 male (BBM).

*Lilioceris laticornis*: Paratype, Hainan: nr. Nodoa, Tan-hsien (Distr.), 17–22.VIII.1928, Lingnan Univ. 5th Hainan Island Expedition, 1 female (USNM).

*Lilioceris maai*: Holotype male: 1) Fukien S. China, Shaowun Tachulan 1000 m. T. Maa; 2) May 7, 1942; 3) Holotype Lilioceris maai J. L. Gressitt + Kim; 4) Lilioceris maai Gres. Kim. J. L. Gressitt det.; 5) Lilioceris sp. 1 maai; 6) Lilioceris impressa (F.) det. A. Konstantinov 2010. (BBM). Paratypes CHINA. Fujian: Shaowu, Tachulan, 26.IV.1942, T.C. Maa, 2 females (BBM), same locality and collector, but 1000m, 7.6.1943 female (BBM); same locality and collector, but 17.V.1942, 1 female (USNM); same locality, but 17.V.1945, K. C. Lin, 1 female (BBM).

*Lilioceris ruficornis*, Lectotype, female. Labels. 1)“Pe yen Tsing Yunnan; 2) type; 3) TYPE; 4) *ruficornis n sp*; 5) Lectotype Crioceris ruficollis Pic des Konstantinov and Tishechkin 2010 (MNHN).

*Lilioceris subcostata*, Lectotype, male. Labels. 1) Shin-Guy-Foo CHINE ; 2) octobre; 3) n sp; 4) type; 5) TYPE; 6) *subcostata n sp*; 7) Lectotype Crioceris subcostata Pic des Konstantinov and Tishechkin 2010 (MNHN).

#### Material examined.

 **CHINA**. **Hainan:** Dwa Bi, 20.VII. 1935, L. Gressitt, identified as *Lilioceris laticornis* by S. Kimoto, 1959, 1 female (BBM); same locality, but 22.VII.1935 (1 BBM); Pan Heang 8.VI.1935, L. Gressitt (1 BBM). **Sichuan:** between Yachow and Kiating, 19–22.VI.1929, 1200–1500 feet, D. C. Graham, 1 specimen (USNM). **Yunnan:** Pe Yen Tsing, 1 specimen (USNM); Tche-Ping-Tcheou, 8 specimens (USNM); no precise locality, 1 specimen (USNM). **INDIA. Haryana:** Kalka, 1 specimen (USNM). **LAOS.** **Sayaboury Prov.** Sayaboury 30.V.1065, (1 BBM); **Khammouane Prov**. Phon Tiou, 6.VII.1965 (3 BBM). **MALAYSIA.** Malacca, 1 specimen (USNM). **THAILAND.** Trong, lower Siam, W. L. Abbott, 2 specimens (USNM). **VIETNAM.** Hanoi, 24.XII.1961, at lights, O. N. Kabakov, 1 specimen (USNM); Trang Bom, 30 mi NW of Saigon, 18.VII.1932, M. Poilane, 1 specimen (USNM); same locality and collector, but 26.VII.1932, 1 specimen (USNM); Nord Annam, Than-Hoa, V.1942, R. Dessom, 1 specimen (USNM); mountains 50 km NE Thai Nguen, 300 m, 13.IX.1962, O. N. Kabakov, 1 specimen (USNM).

**Figures 10–13.**
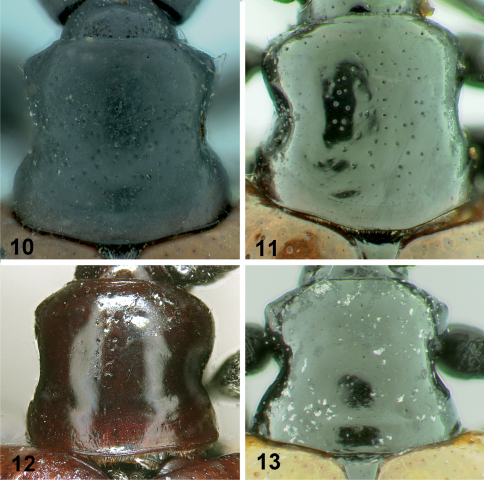
Pronotums of *Lilioceris* species, dorsal view. **10** *Lilioceris impressa* **11** *Lilioceris laosensis* **12** *Lilioceris unicolor* **13** *Lilioceris yunnana*.

### Lilioceris laosensis

(Pic, 1916)

http://species-id.net/wiki/Lilioceris_laosensis

Figs 11 17, 22 27

Crioceris laosensis Pic, 1916:16 (Type locality: “Laos”. Lectotype designated here, MNHN). Kimoto and Gressitt, 1979:227 (resurrected from synonymy).

#### Diagnosis.

 Occipital area with a shallow furrow, at least with a deep small fovea at midpoint. Pronotum posteriorly with two weak, but distinct transverse irregular impressions. Pronotal disc with larger, scattered punctures, mid-line alignment of punctures usually still evident, at least in anterior half. Lateral sides of pronotum around constriction with large punctures. Outer metasternal disc mostly free of setae. Anterior setose fringes not expanded in antero-lateral corners. At least half of metepisternal disc covered with setae.

Apical elytral punctures weakened. Apical elytral intervals not raised. Internal sac of aedeagus with posterior part of dorsal sclerite in dorsal view more or less widely triangular, directed slightly medially. Posterior part of dorsal sclerite in lateral view more or less widely triangular, directed forward.

#### Type material examined.

 *Lilioceris laosensis*: Lectotype, male. Labels: 1) TAKEK LAOS COLL. LE MOULT; 2) [illegible] roux; 2) type; 3) TYPE; 4) *laosensis* Pic; 5)Lectotype *Lilioceris laosensis* Pic A. Tishechkin & A. Konstantinov des. 2010” (MNHN); paralectotype, male, the same labels as lectotype (MNHN).

#### Material examined.

 **CHINA**. **Fujian:** near Foochow, 1921, C. R. Kellogg, 1 specimen (USNM); **Yunnan**: Ma-Chang, 1000 m, 1 specimen (USNM); Tche-Ping-Tcheou, 1 specimen (USNM). **INDIA.** Inde or, 1 specimen (USNM). **Assam:** Chabua, 8.V.1944, G. Butler, 4 specimens (USNM); Doom Dooma, VI.1943, D. E. Hardy, 1 specimen (USNM). **Karnataka**: Malabar, 1 specimen (USNM); Western Ghats, 7 km N Chickmagular, 13º23’23”N, 75º42’9”E, 1800 m, 15.XI.2003, A. Konstantinov, K. Prathapan & S. Saluk, 1 specimen (USNM). **Sikkim:** Rungbong Vall., Gopaldhara, 1916, H. Stevens, 1 specimen (MNHN); without precise locality, IX.1957, G. W. Angalet, 1 specimen (USNM). **LAOS.** **Borikhane Prov.** Pakkading 29.IV.1966 (1 BBM). **MYANMAR.** Carin Cheba, 1000 m, V.1888, L. Fea, 2 specimens (USNM); Tenasserim, Meetan, IV.1887, L. Fea, 1 specimen (USNM). **NEPAL.** Jiri Reg., Shivalaya-Jiri, 27º36.61’N, 86º17.55’E, 1770–1900 m, pass, 2200m, 12.V.2000, A. Konstantinov, S. Lingafelter & M. Volkovitch, 1 specimen (USNM); Sankhua Sabha Dist., Arun Valley, Chichila, 1900–2000 m, 18–20.VI.1988, J. Martens & W. Schawaller, 1 specimen (USNM). **THAILAND.** Doi Sutep, 26.VIII.1951, 1 specimen (USNM); Trong, lower Siam, W. L. Abbott, 4 specimens (USNM). **VIETNAM.** Bao Lac (Tonkin), 2 specimens (USNM); Nord Annam, Than-Hoa, V.1942, R. Dessom, 1 specimen (USNM); Trang Bom, 30 mi NW of Saigon, 23.VII.1932, M. Poilane, 1 specimen (USNM).

**Figures 14–22.**
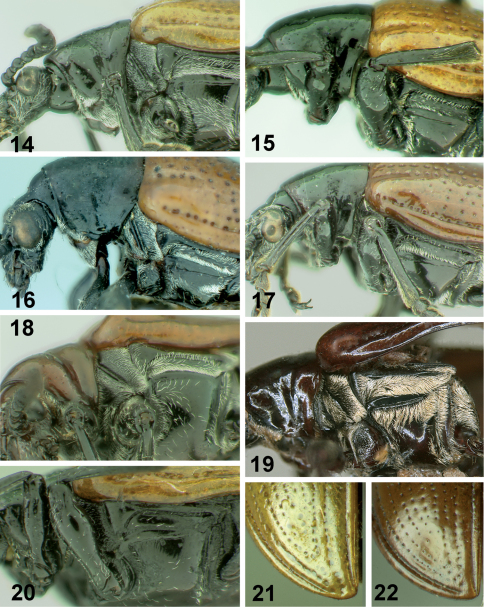
Thorax of *Lilioceris* species, **14–20** lateral view; **21, 22** elytral apex. **14** *Lilioceris cheni* **15** *Lilioceris egena* **16** *Lilioceris impressa* **17** *Lilioceris laosensis* **18** *Lilioceris malabarica* **19** *Lilioceris unicolor* **20** *Lilioceris yunnana* **21** *Lilioceris cheni* **22** *Lilioceris laosensis*.

### Lilioceris malabarica

(Jacoby, 1904)

http://species-id.net/wiki/Lilioceris_malabarica

Figs 18 28

Crioceris malabarica Jacoby, 1904:381 (Type locality: “Malabar”. Lectotype designated here, BMNH).

#### Diagnosis.

 Occipital area with a shallow furrow, at least with a deep small fovea at midpoint. Pronotum posteriorly with single weak, regular, transverse impression. Pronotal disc without or with few small punctures, larger punctures present only along pronotal mid-line, at least in anterior half. Lateral sides of pronotum around constriction impunctate. Outer metasternal disc mostly free of setae. Anterior setose fringes expanded in antero-lateral corners. At least half of metepisternal disc covered with setae. Apical elytral punctures weakened. Apical elytral intervals not raised. Internal sac of aedeagus with posterior part of dorsal sclerite in dorsal view more or less widely triangular, directed forward. Posterior part of dorsal sclerite in lateral view more or less widely triangular, directed forward.

#### Type material examined.

 *Lilioceris malabarica*: Lectotype, male. Labels: 1) Type H.T.; 2) 432; 3) Mahe Malabar; 4) Jacoby Coll. 1909-28a; 5) Crioceris malabarica Jac.; 6) Lectotype Lilioceris malabarica (Jacoby), des. Konstantinov and Tishechkin 2010 (BMNH). Paralectotype, female, same label as lectotype (BMNH). Paralectotype, male. Labels: 1) Nilgiri Hills; 2) 810; 3) Crioceris semipunctata Fab.; 4) Crioceris malabarica HEA Jac; 5) Andrewes Bequest B.M. 1922-221; 6) Paralectotype Lilioceris malabarica (Jacoby), des. Konstantinov and Tishechkin 2010 (BMNH).

#### Material examined.

 **INDIA. Kerala:** Mahé, Malabar, 1 specimen (USNM); Malabar, gift of F. C. Bowditch, 4 specimens (USNM).

**Figures 23–25.**
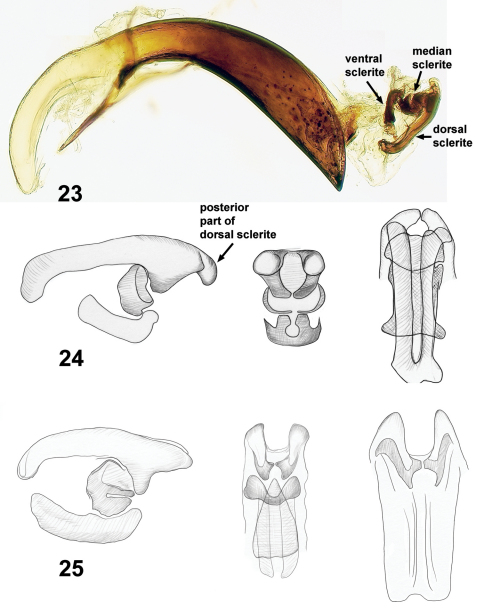
Aedeagi of *Lilioceris* species, **23** aedeagus in lateral view with internal sac everted **24–25** sclerites of internal sac. **23, 24** *Lilioceris cheni* (lateral, frontal and dorsal views) **25** *Lilioceris egena* (lateral, frontal and dorsal views).

### Lilioceris thibetana

(Pic, 1916)

http://species-id.net/wiki/Lilioceris_thibetana

Figs 4 29

Crioceris thibetana Pic, 1916:18 (Type locality: “Thibet”. Lectotype designated here, MNHN). Gressitt and Kimoto, 1961:59 (valid species). Kimoto and Gressitt 1979:229 [synonymy with *Lilioceris laosensis* (Pic)]. Status resurrected

#### Diagnosis.

 Occipital area with a deep small fovea at midpoint. Pronotum posteriorly with single weak, regular, transverse impression. Lateral sides of pronotum around constriction with large punctures. Pronotal disc with scattered larger punctures, mid-line alignment of punctures usually still evident, at least in anterior half. Outer metasternal disc mostly free of setae. Anterior setose fringes not expanded in antero-lateral corners. About half of metepisternal disc covered with setae. Apical elytral punctures strong. Apical elytral intervals not raised. Internal sac of aedeagus with posterior part of dorsal sclerite in dorsal view very short, widely rounded, directed laterally. Posterior part of dorsal sclerite in lateral view very short, widely rounded, directed forward.

#### Type material examined.

 *Lilioceris thibetana*: Lectotype, male. Labels: 1) Thibet Tianatung; 2) n sp; 3) type; 4) TYPE; 5) thibetana Pic; 6) Lectotype Crioceris thibetana Pic des. A. Konstantinov & A. Tishechkin 2010 (MNHN).

**Figures 26–28.**
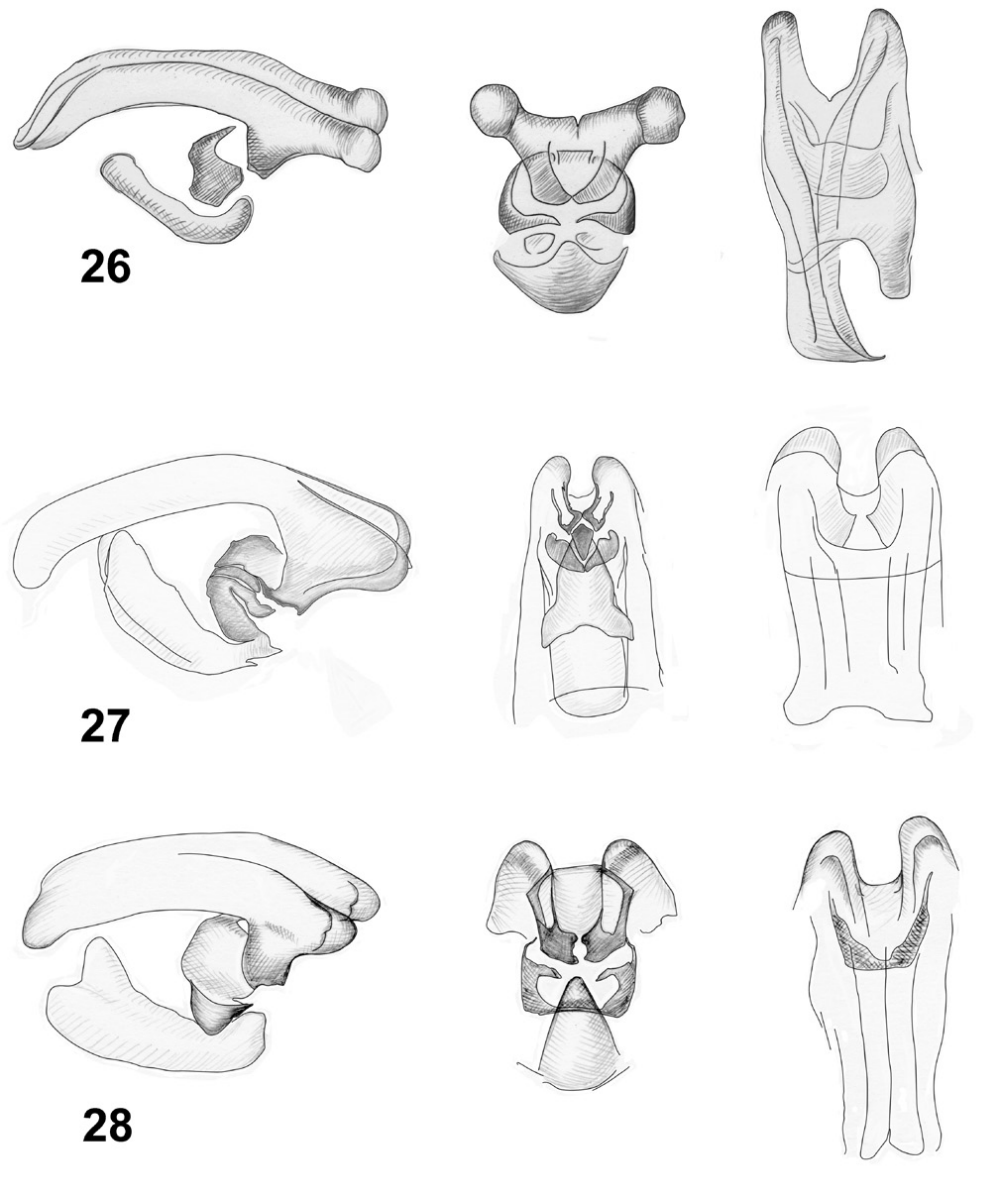
Internal sacs of aedeagi of *Lilioceris* species (lateral, frontal and dorsal views), **26** *Lilioceris impressa* **27** *Lilioceris laosensis* **28** *Lilioceris malabarica*.

### Lilioceris unicolor

(Hope, 1831)

http://species-id.net/wiki/Lilioceris_unicolor

Figs 5 12 19 30

Crioceris unicolor Hope, 1831:28 (Type locality: Nepal. Lectotype designated here, BMNH)
Crioceris badia Lacordaire, 1845:560 (Type locality: Siam. Type, not found). Clavareau (1913) (synonymy).

#### Diagnosis.

 Occipital area with shallow but sharp furrow. Pronotum posteriorly with single weak, regular, transverse impression. Pronotal disc with small punctures situated in middle in two rows. Lateral sides of pronotum around constriction without punctures. Outer metasternal disc only with oblique setose patch or covered with dense setae along its lateral side. Anterior setose fringes expanded in antero-lateral corners, which are covered with dense yellow setae. Metepisternal disc completely covered with setae. Apical elytral punctures weak. Apical elytral intervals flattened. Internal sac of aedeagus with posterior part of dorsal sclerite in dorsal view with two apices, lateral longer than median, directed medially. Posterior part of dorsal sclerite in lateral view more or less parallel-sided, slightly swollen at apex, directed dorsally.

#### Comments.

 We were not able to find the type of *Lilioceris badia*, but found a specimen in the BMNH from the Baly collection with a label identifying it as *Lilioceris badia*. The specimen is conspecific with *Lilioceris unicolor*.

#### Type material examined.

 *Lilioceris unicolor*: Lectotype, male. Labels: 1) Nepal; 2) Type; unicolor Hope; 3) Hardwicke Bequest; 4) Lectotype Lilioceris unicolor Hope des. A. Konstantinov & A. Tishechkin 2010 (BMNH).

#### Material examined.

 *Lilioceris badia*: Labels: 1) “illegible” 2) Baly Coll.; 3) Crioceris badia Lac. Siam, “illegible” (BMNH).

### Lilioceris yunnana

(Weise, 1913)

http://species-id.net/wiki/Lilioceris_yunnana

Figs 13 20 31

Crioceris crassicornis Fairmaire, 1887: 136 (Type locality: “Yunnan”. Type depository: unknown) nec Olivier 1808.
Crioceris yunnana Weise, 1913:220 [new name for *Lilioceris crassicornis* (Fairmaire, 1887) nec Olivier 1808].

#### Diagnosis.

 Occipital area with a shallow furrow, at least with a deep small fovea at midpoint. Pronotum posteriorly more or less convex, no distinct impressions present. Pronotal disc without or with few small punctures, larger punctures present only along pronotal mid-line, at least in anterior half. Lateral sides of pronotum around constriction impunctate. Outer metasternal disc mostly free of setae. Anterior setose fringes of metasternum not expanded in antero-lateral corners. Setae on metepisternae occupy only narrow line along inner margin. Apical elytral punctures weakened. Apical elytral intervals not raised. Internal sac of aedeagus with posterior part of dorsal sclerite in dorsal view wide and short, slightly triangular, directed forward. Posterior part of dorsal sclerite in lateral view widely triangular with dorsal side longer than ventral, directed forward, but at the same time posteroventrally.

#### Comments.

 We were unable to find the type of *Lilioceris crassicornis* or specimens of it identified by Weise. We based our concept of this species on the 3 specimens from HUB identified by Heinze.

#### Material examined.

 **CHINA. Hubei:** Lichuan Distr., Suisapa, 1000 m, 21.VIII.1948, J. L. Gressit, 1 specimen (BBM); Lichuan Distr., Leong-ho-kow, 1000 m, 7.IX.1948, Gressit & Djou, 1 specimen (BBM). **Sichuan:** W of Yachou, 16–20.VI.1923, D. C. Graham, 1 specimen (USNM). **Yunnan:** env. Xiaguan, 25º26’29”N, 100º12’51”E, 1821 m, A. Konstantinov & M. Volkovitch, 1 specimen (USNM); Yunnan, 3 specimens (HUB). **INDIA. Sikkim:** Hou He, 1 specimen (USNM). **VIETNAM.** Nord Annam, Than-Hoa, V.1942, R.Dessom, 1 specimen (USNM); Cochinchine, 1 specimen (USNM).

**Figures 29–31.**
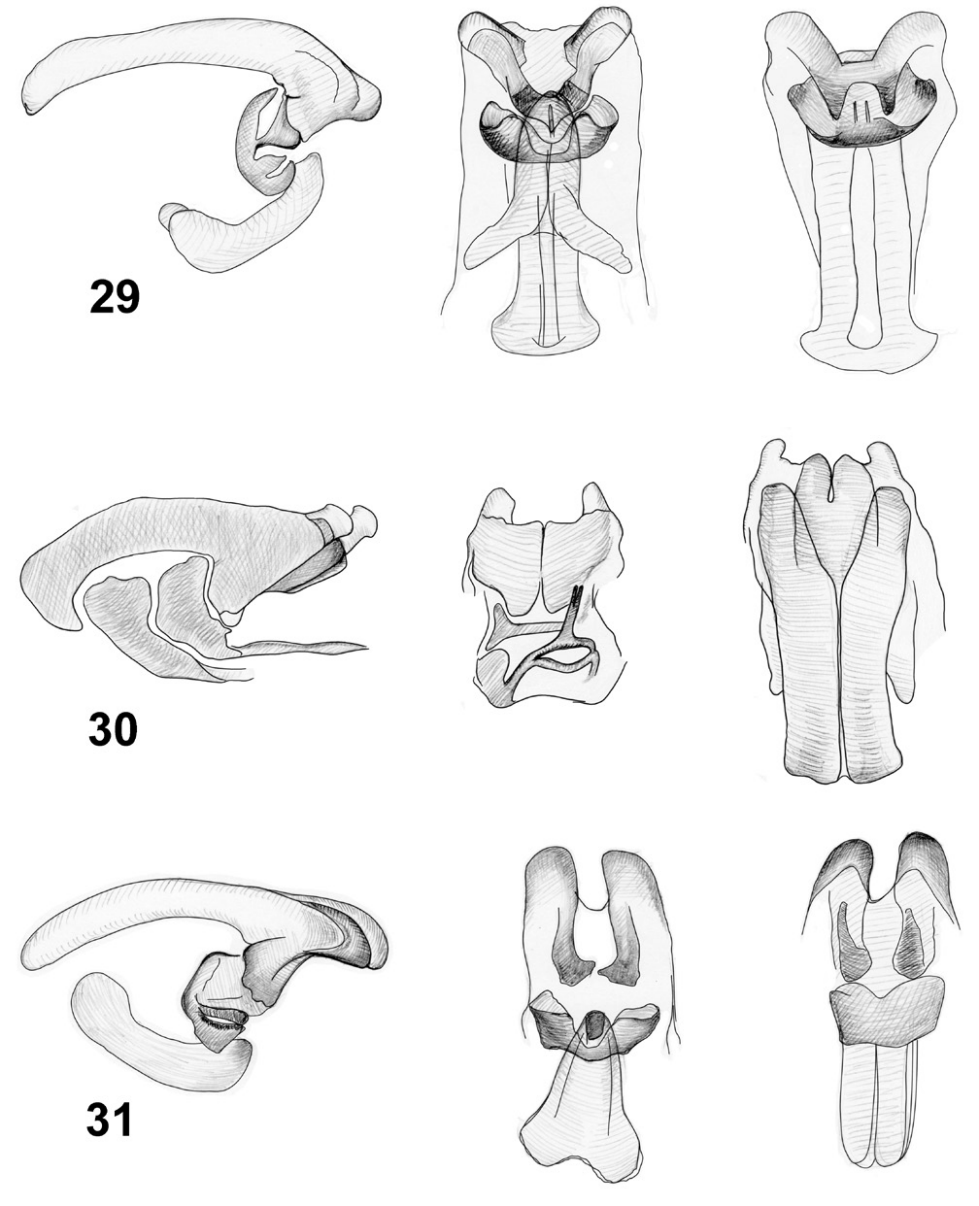
Internal sacs of aedeagi of *Lilioceris* species (lateral, frontal and dorsal views),**29** *Lilioceris thibetana* **30** L*. unicolor* **31** *Lilioceris yunnana*.

## Supplementary Material

XML Treatment for Lilioceris cheni

XML Treatment for Lilioceris egena

XML Treatment for Lilioceris impressa

XML Treatment for Lilioceris laosensis

XML Treatment for Lilioceris malabarica

XML Treatment for Lilioceris thibetana

XML Treatment for Lilioceris unicolor

XML Treatment for Lilioceris yunnana
